# Associations of habitual glucosamine supplementation with incident gout: a large population based cohort study

**DOI:** 10.1186/s13293-022-00461-z

**Published:** 2022-09-30

**Authors:** Mengyi Liu, Ziliang Ye, Yanjun Zhang, Sisi Yang, Qimeng Wu, Chun Zhou, Panpan He, Yuanyuan Zhang, Xiaoqin Gan, Xianhui Qin

**Affiliations:** Division of Nephrology, Nanfang Hospital, Southern Medical University, National Clinical Research Center for Kidney Disease, State Key Laboratory of Organ Failure Research, Guangdong Provincial Institute of Nephrology, Guangdong Provincial Key Laboratory of Renal Failure Research, Guangzhou, 510515 China

**Keywords:** Glucosamine, Gout, Females, Diuretics, Genetic risk scores

## Abstract

**Objectives:**

The association between habitual glucosamine use and incident gout has not been examined in previous studies. We aimed to evaluate the association of habitual use of glucosamine with the risk of gout in general population.

**Methods:**

A total of 436,594 participants (55.4% female) without prior gout at baseline who completed a questionnaire on supplementation use, which included glucosamine, in the UK Biobank were enrolled. Incident gout was recorded from self-report, death register, primary care, and hospital admission data.

**Results:**

At baseline, 53,433 (22.1%) females and 30,685 (15.8%) males reported habitual glucosamine use. During a median follow-up period of 12.1 years, 1718 (0.7%) females and 5685 (2.9%) males developed gout. After multivariable adjustment for major risk factors, glucosamine use was associated with a significantly lower risk of incident gout in females (hazard ratio [HR], 0.81, 95% confidence interval [CI], 0.71–0.92), but not in males (HR, 1.05, 95% CI, 0.97–1.13), compared with non-use (*P*-interaction < 0.001). Among females, the inverse association between glucosamine use and gout was stronger in participants with diuretics use (HR, 0.64, 95% CI, 0.50–0.81) than those without diuretics use (HR, 0.89, 95% CI, 0.77–1.03) (*P*-interaction = 0.015). Moreover, gout genetic risk scores did not significantly modify the association between glucosamine use and the risk of incident gout in males (*P*-interaction = 0.548) or females (*P*-interaction = 0.183).

**Conclusions:**

Habitual glucosamine use to relieve osteoarthritis pain was related to lower risk of gout in females, but not in males.

**Supplementary Information:**

The online version contains supplementary material available at 10.1186/s13293-022-00461-z.

## Highlights

1. Glucosamine use was associated with lower risk of incident gout in females.
2. The inverse association between glucosamine use and gout was stronger in females with diuretics use.
3. Gout genetic risk scores did not significantly modify the association.

**Supplementary Information:**

The online version contains supplementary material available at 10.1186/s13293-022-00461-z.

## Introduction

Gout, an inflammatory crystal-induced arthritis caused by the deposition of monosodium urate crystals in articular and non-articular structures, affects approximately 41.2 million adults worldwide [1, 2]. The effects of gout include not only acute pain, functional disability, and permanent joint damage, but also significant long-term illness and costs [3, 4]. Due to the increasing prevalence and incidence of gout, as well as the suboptimal management of gout in many countries [5], identifying more effective, safe and economical primary prevention measures has important clinical value.

Glucosamine is a popular non-vitamin, non-mineral dietary supplement widely used to relieve osteoarthritis and joint pain [6, 7], with a high safety profile, and has recently garnered interest for their potential anti-inflammatory effects [7]. Inflammation plays an essential role in gout [8]. Accordingly, a recent study found that the IL-1β inhibitor canakinumab administration was associated with significantly reduced risk for gout attacks in patients with a prior myocardial infarction [9]. Therefore, given the anti-inflammatory properties of glucosamine, we hypothesized that habitual glucosamine use may be also related to decreased gout risk. However, no studies have analyzed the association between habitual glucosamine use and incident gout in prospective cohorts.

To address these aforementioned gaps in knowledge, the present study aimed to evaluate the association of habitual use of glucosamine with the risk of gout in general population, using population-based cohort data from nearly half a million adults in the UK Biobank study. We also assessed the joint association of glucosamine use and genetic susceptibility with the risk of gout.

## Methods

### Data source and study population

The UK Biobank is a large prospective, observational, population-based cohort of half a million adult residents of the United Kingdom, aged 37–73 years, from 22 assessment centers across England, Wales, and Scotland between 2006 and 2010. Participants were asked to complete a touch screen questionnaire, a face-to-face interview and a series of physical measurements, as well as provide biological samples for laboratory analysis. The details of the study design have been described previously [10, 11]. The UK Biobank was approved by the North West Research Ethics Committee (06/MRE08/65) and all participants signed an informed consent.

In this study, we restricted our analysis to participants who had complete information on the use of glucosamine and were free of gout (*n* = 484,720). We also excluded participants with unavailable genetic data and data on the important covariates (*n* = 48,126). Therefore, a total of 436,594 participants were enrolled in the present analysis (Additional file 1: Fig. S1).

### Ascertainment of exposure and covariates

At baseline, habitual glucosamine information was collected through a touch-screen questionnaire. Participants were asked, “Do you regularly take any of the following?” and could select their answer from a list of supplementations, including vitamin, mineral, fish oil and glucosamine. From this information, regular use of glucosamine was defined as “1 = yes” and “0 = no”.

Detailed information on covariates was available through standardized questionnaires, including age, sex, race, Townsend Deprivation Index (TDI), smoking status, alcohol consumption, comorbidities (hypertension, diabetes, high cholesterol, osteoarthritis, rheumatoid arthritis, and joint pain), and drug use (cholesterol lowering medication, anti-hypertensive drug, insulin, aspirin, ibuprofen, paracetamol, and diuretics). Body mass index (BMI) (kg/m^2^) was calculated based on measured weight and height. Prevalent diabetes at baseline was identified through multiple procedures considering type of diabetes and sources of the diagnosis [12]. A healthy diet score was evaluated using a more recent dietary recommendation for cardiovascular health, which considered adequate consumption of fruit, vegetables, whole grains, fish, shellfish, dairy products, and vegetable oils and reduced consumption of refined grains, processed meats, unprocessed meats, and sugar sweetened beverages, and a healthy diet was defined as meeting at least five items of the recommendations [13]. In addition, biochemistry measures were performed at a dedicated central laboratory, including creatinine, urate and C-reactive protein (CRP). Estimated glomerular filtration rate (eGFR) was calculated by Chronic Kidney Disease–Epidemiology Collaboration equation (CKD–EPI) using serum creatinine [14].

### Definition of genetic risk score

Detailed information about genotyping and quality control in the UK Biobank study has been described previously [15]. We selected 13 single nucleotide polymorphisms (SNPs) which showed independently significant genome-wide association with gout in recent published genome-wide associations studies (Additional file 1: Table S1) [16]. Genetic risk score (GRS) was calculated using a weighted method [17] and a higher score indicates a higher genetic predisposition to gout, and participants were divided into low, intermediate, or high genetic risk for gout according to the tertiles of GRS.

### Ascertainment of outcomes

The primary outcome of the study was the incidence of gout, and gout diagnosis was extracted from “first occurrence of health outcomes defined by a 3-character International Statistical Classification of Diseases and Related Health Problems 10th Revision code (M10)” based on self-report or linkage to death register and/or primary care and/or hospital admission data. The follow-up person-time for each participant was calculated from the date of first assessment until the date of death, first date of outcome diagnosis, date of lose to follow-up, or end of follow-up, whichever came first.

### Statistical analysis

Population characteristics are presented as mean ± standard deviation (SD) for continuous variables and proportions for categorical variables. Comparisons of characteristics according to glucosamine use (yes or no) by sex were performed by chi-square tests for categorical variables and *t* tests for continuous variables.

Cox proportional hazards models were used to estimate hazard ratio (HR) and 95% confidence interval (CI) of gout for habitual glucosamine use (yes vs. no). The proportional hazard assumption was evaluated by the interaction between exposures and follow-up time and no violation of this assumption was detected. In multivariable models, potential confounders that were known to be traditional or suspected risk factors for gout were adjusted for, including age, sex, race, TDI, BMI, smoking status, alcohol consumption, healthy diet score, vitamin or mineral supplementation, fish oil supplementation, comorbidities (hypertension, diabetes, high cholesterol, osteoarthritis, rheumatoid arthritis, and joint pain), drug uses (cholesterol lowering medication, anti-hypertensive drug, insulin, aspirin, ibuprofen, paracetamol, and diuretics), eGFR, and urate. To control the potential influence of genetic predisposition to gout, we further adjusted for gout GRS, as well as estimated the joint association of glucosamine use and gout GRS with the risk of incident gout using glucosamine non-users with low genetic risk as reference.

Stratified analysis was conducted to assess potential modification effects of glucosamine use according to age (< 60 or ≥ 60 years), BMI (< 30 or ≥ 30 kg/m^2^), smoking status (never or ever), alcohol consumption (< 1 or ≥ 1 times/week), health diet (yes or no), supplementation use (yes or no), diabetes (yes or no), hypertension (yes or no), diuretics use (yes or no), aspirin use (yes or no), paracetamol or ibuprofen use (yes or no), and CRP (tertiles). Potential modifying effects were assessed by modelling the cross product term of the stratifying variable with glucosamine use.

A two-tailed *P* < 0.05 was considered to be statistically significant in all analyses. Analyses were performed using R 4.1.1 software (http://www.R-project.org/).

## Results

### Study participants and baseline characteristics

Among 436,594 participants in the current study, 242,009 (55.4%) were females, with a mean age of 56.5 years. Overall, 53,433 (22.1%) females and 30,685 (15.8%) males reported habitual glucosamine supplementation at baseline.

As shown in Table 1, compared with nonusers, glucosamine users were older, more likely to be White, non-current smokers, dietary supplementation users, and tended to have lower TDI, higher alcohol intake and healthy diet, higher prevalence of high cholesterol, osteoarthritis and joint pain, but a lower prevalence of diabetes, as well as more likely to take aspirin, ibuprofen, and paracetamol. In addition, glucosamine users tended to have higher prevalence of hypertension and more likely to take antihypertensive drugs among females, while glucosamine users tended to have lower prevalence of hypertension and less likely to take antihypertensive drugs among males.

**Table 1:** Baseline population characteristics according to glucosamine use

	Females			Males		
	Glucosamine non-users	Glucosamine users	*P* value	Glucosamine non-users	Glucosamine users	*P* value
*N*	188,576	53,433		163,900	30,685	
Age, years	55.6 ± 8.1	59.1 ± 6.8	< 0.001	56.2 ± 8.3	58.9 ± 7.5	< 0.001
White, No. (%)	178,685 (94.8)	51,366 (96.1)	< 0.001	155,205 (94.7)	29,721 (96.9)	< 0.001
Townsend Deprivation Index	− 1.3 ± 3.1	− 1.8 ± 2.8	< 0.001	− 1.2 ± 3.2	− 1.9 ± 2.8	< 0.001
Body mass index, kg/m^2^	27.0 ± 5.2	27.0 ± 4.9	0.659	27.7 ± 4.2	27.7 ± 3.9	0.609
Smoking status, No. (%)			< 0.001			< 0.001
Never	112,451 (59.6)	31,753 (59.4)		81,245 (49.6)	14,921 (48.6)	
Former	57,705 (30.6)	18,682 (35.0)		60,918 (37.2)	13,344 (43.5)	
Current	18,420 (9.8)	2998 (5.6)		21,737 (13.3)	2420 (7.9)	
Alcohol consumption, times/week			< 0.001			< 0.001
< 1	72,232 (38.3)	17,561 (32.9)		38,058 (23.2)	5497 (17.9)	
1–2	48,838 (25.9)	13,712 (25.7)		43,131 (26.3)	7605 (24.8)	
3–4	38,116 (20.2)	12,086 (22.6)		42,314 (25.8)	8870 (28.9)	
> 4	29,390 (15.6)	10,074 (18.9)		40,397 (24.6)	8713 (28.4)	
Healthy diet, No. (%)	52,379 (27.8)	17,976 (33.6)	< 0.001	31,650 (19.3)	7749 (25.3)	< 0.001
Vitamin and mineral supplementation, No. (%)	63,844 (33.9)	33,385 (62.5)	< 0.001	38,187 (23.3)	17,238 (56.2)	< 0.001
Oily fish supplementation, No. (%)	46,324 (24.6)	33,114 (62.0)	< 0.001	38,154 (23.3)	19,864 (64.7)	< 0.001
Disease history, No. (%)						
Diabetes	6777 (3.6)	1332 (2.5)	< 0.001	11,155 (6.8)	1481 (4.8)	< 0.001
Hypertension	43,183 (22.9)	12,660 (23.7)	< 0.001	47,857 (29.2)	8667 (28.2)	< 0.001
High cholesterol	17,959 (9.5)	5537 (10.4)	< 0.001	23,947 (14.6)	4628 (15.1)	0.032
Osteoarthritis	13,450 (7.1)	10,028 (18.8)	< 0.001	7503 (4.6)	4159 (13.6)	< 0.001
Rheumatoid arthritis	2652 (1.4)	774 (1.4)	0.466	1123 (0.7)	264 (0.9)	< 0.001
Joint pain	550 (0.3)	344 (0.6)	< 0.001	426 (0.3)	197 (0.6)	< 0.001
Drugs use, No. (%)						
Antihypertensive	32,608 (17.3)	9533 (17.8)	0.003	38,903 (23.7)	6879 (22.4)	< 0.001
Cholesterol-lowering	23,446 (12.4)	6969 (13.0)	< 0.001	36,307 (22.2)	6834 (22.3)	0.644
Insulin	1640 (0.9)	266 (0.5)	< 0.001	2434 (1.5)	299 (1.0)	< 0.001
Aspirin	18,147 (9.6)	5774 (10.8)	< 0.001	30,099 (18.4)	5945 (19.4)	< 0.001
Ibuprofen	31,174 (16.5)	10,834 (20.3)	< 0.001	18,075 (11.0)	5133 (16.7)	< 0.001
Paracetamol	49,286 (26.1)	14,972 (28.0)	< 0.001	27,481 (16.8)	5585 (18.2)	< 0.001
Diuretics	13,377 (7.1)	4090 (7.7)	< 0.001	10,900 (6.7)	1942 (6.3)	0.037
Estimated glomerular filtration rate, mL/min/1.73 m^2^	91.5 ± 13.6	89.5 ± 12.4	< 0.001	91.1 ± 13.3	89.7 ± 12.0	< 0.001
Urate, umol/L	270.3 ± 66.2	271.5 ± 64.2	< 0.001	353.2 ± 70	349.9 ± 66.5	< 0.001

Values are presented as means ± SD or proportions

### Association between glucosamine use and risk of incident gout

During a median follow-up period of 12.1 years (5,124,432 person-years), a total of 7403 (1.7%) participants, including 1718 (0.7%) females and 5685 (2.9%) males, developed gout.

After adjustment for important covariates including socioeconomic and behavioral factors, comorbidities, and drug uses, glucosamine use was associated with lower risks of gout in all participants (HR, 0.87, 95% CI, 0.81–0.93), females (HR, 0.75, 95% CI, 0.66–0.85), and males (HR, 0.92, 95% CI, 0.85–0.99) (Table 2). However, when further adjusted for eGFR and urate, the association only persisted in females (HR, 0.81, 95% CI, 0.71–0.92), but not in males (HR, 1.05, 95% CI, 0.97–1.13) (*P*-interaction < 0.001). Further adjustment for gout GRS did not materially change the magnitude and the significance (Table 2).

**Table 2:** Relationship of glucosamine uses with risk of incident gout

	Total population		Females		Males	
	Glucosamine non-users	Glucosamine users	Glucosamine non-users	Glucosamine users	Glucosamine non-users	Glucosamine users
Total	352,476	84,118	188,576	53,433	163,900	30,685
No of events	6156	1247	1375	343	4781	904
Incidence rates*	1.5	1.3	0.6	0.5	2.5	2.5
Crude model	ref	0.84(0.79, 0.90)	ref	0.88(0.78, 0.99)	ref	1.01(0.94, 1.08)
Adjusted model 1^†^	ref	0.87(0.81, 0.93)	ref	0.75(0.66, 0.85)	ref	0.92(0.85, 0.99)
Adjusted model 2^†^	ref	0.97(0.91, 1.04)	ref	0.81(0.71, 0.92)	ref	1.05(0.97, 1.13)
Adjusted model 3^†^	ref	0.97(0.91, 1.04)	ref	0.81(0.71, 0.92)	ref	1.05(0.97, 1.13)

^*^Incidence rates per 1000 person years

^**†**^Adjusted Model 1: adjusted for age, sex (only for total population), race, Townsend Deprivation Index, body mass index, smoking status, alcohol consumption, healthy diet score, vitamin or mineral supplementation, fish oil supplementation, comorbidities (hypertension, diabetes, high cholesterol, osteoarthritis, rheumatoid arthritis, and joint pain), and drug use (cholesterol lowering medication, anti-hypertensive drug, insulin, aspirin, ibuprofen, paracetamol, and diuretics); adjusted model 2: adjusted for the covariates in Model 1 and further adjusted for estimated glomerular filtration rate and urate; adjusted model 3: adjusted for the covariates in Model 2 and further adjusted for gout genetic risk score

### Joint association of glucosamine use and gout GRS with the risk of incident gout

**Fig. 1:**
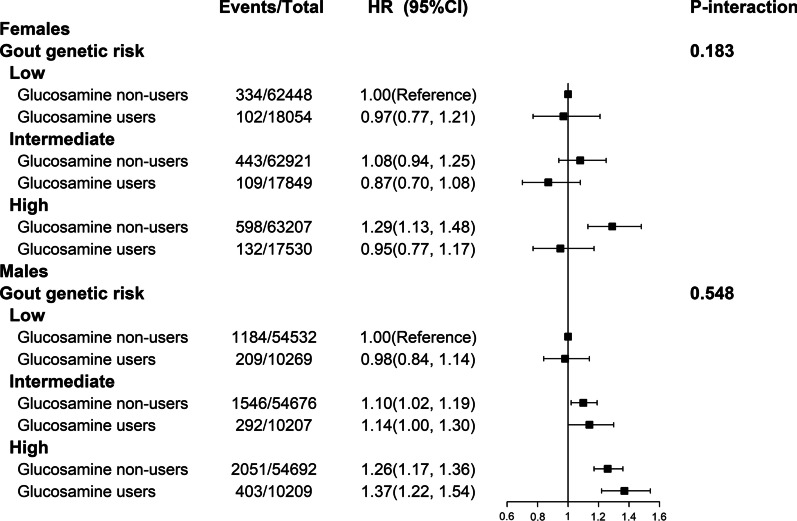
Joint association of glucosamine use and genetic risk in relation to risk of gout. Adjusted for age, sex (only for total population), race, Townsend Deprivation Index, body mass index, smoking status, alcohol consumption, healthy diet score, vitamin or mineral supplementation, fish oil supplementation, comorbidities (hypertension, diabetes, high cholesterol, osteoarthritis, rheumatoid arthritis, and joint pain), drug use (cholesterol lowering medication, anti-hypertensive drug, insulin, aspirin, ibuprofen, paracetamol, and diuretics), estimated glomerular filtration rate and urate

In the joint analysis, as expected, higher gout GRS was significantly associated with higher risk of incident gout (Fig. 1).

However, gout GRS did not significantly modify the association between glucosamine use and the risk of incident gout in males (*P*-interaction = 0.548) or females (*P*-interaction = 0.183). Therefore, among females, glucosamine non-users with a high genetic risk had the highest risk of gout (HR, 1.29, 95% CI, 1.13–1.48), compared with glucosamine non-user with a low genetic risk (Fig. 1).

### Stratified analyses

**Fig. 2:**
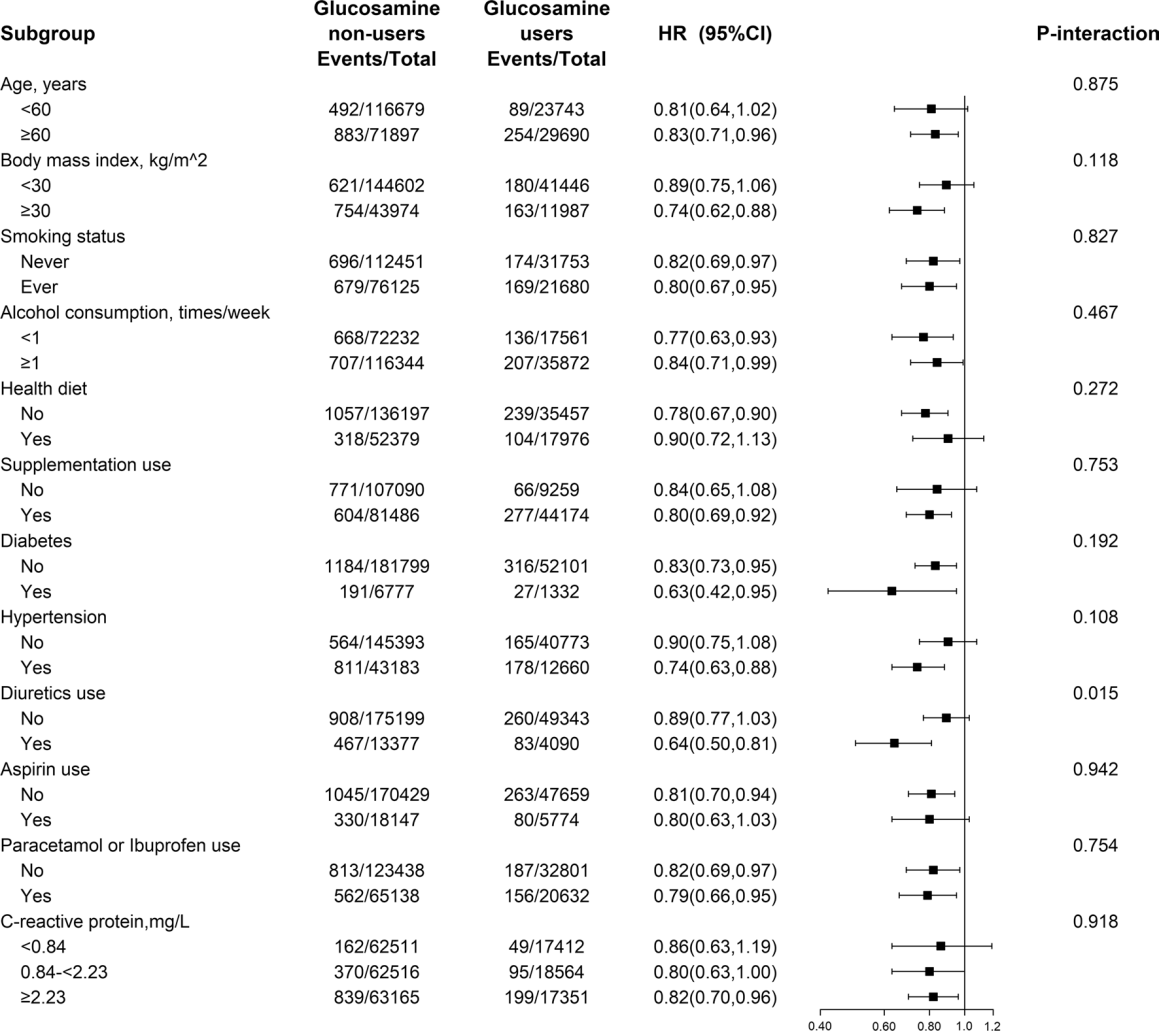
Association of glucosamine use and the risk of gout stratified by potential risk factors in females. Results were adjusted for age, race, Townsend Deprivation Index, body mass index, smoking status, alcohol consumption, healthy diet score, vitamin or mineral supplementation, fish oil supplementation, comorbidities (hypertension, diabetes, high cholesterol, osteoarthritis, rheumatoid arthritis, and joint pain), drug use (cholesterol lowering medication, anti-hypertensive drug, insulin, aspirin, ibuprofen, paracetamol, and diuretics), estimated glomerular filtration rate and urate, if not already stratified

Stratified analyses were separately performed by sex to further assess the relation of regular glucosamine supplementations with the risk of incident gout in various subgroups (Fig. 2).

Among females, the inverse association was stronger with diuretics uses (*P*-interaction = 0.015; Fig. 2). No other significant variables, including age, BMI, smoking status, alcohol consumption, health diet, supplementation use, diabetes, hypertension, diuretics use, aspirin use, paracetamol or ibuprofen use, and CRP showed significant effect modifications on the association of glucosamine use with gout incidence in females (all *P* for interaction ≥ 0.05; Fig. 2). Notably, none of the above variables significantly modified the association between glucosamine use and the risk of incident gout in males (all *P* for interaction ≥ 0.05; Additional file 1: Fig. S2).

## Discussion

In this large population-based cohort of individuals, habitual glucosamine use was associated with a significantly lower risk of incident gout in females but not in males, independent of traditional risk factors and genetic risk factors. Furthermore, the protective associations of glucosamine use with gout risk appeared to be somewhat stronger in females who took diuretics medications.

To the best of our knowledge, the current study is the first one to explore the association of regular glucosamine use with risk of incident gout in a prospective cohort. Based on the UK Biobank cohort, several previous studies have demonstrated that habitual use of glucosamine was associated with lower risk of 17% for incident type 2 diabetes, 15% for cardiovascular disease events, 20% for chronic obstructive pulmonary disease, 15% for all-cause mortality, 18% for mortality from cardiovascular disease, 6% for mortality from cancer, and 27% for mortality from respiratory mortality [18–21]. Consistently, our study first observed 19% lower risk of gout associated with glucosamine use in females. In addition, although the interaction was not statistically significant, the reduced risk with glucosamine appeared to be more pronounced in women with a higher genetic risk.

The precise biological mechanisms underlying the inverse association between glucosamine use and risk of gout in females is not fully understood. Evidences from previous studies have shown that glucosamine has anti-inflammatory properties [22, 23]. Given the detrimental roles of inflammation in the development of gout, we assumed that glucosamine supplementation might reduce the gout risk partly through the anti-inflammatory effect. Interestingly, our findings showed that females are more likely to benefit from glucosamine in terms of risk of gout, which may be partly explained by the sex differences in inflammation. For example, estrogen can either promote inflammation by enhancing Th1 and/or Th17 responses though transcriptional activation of NFκB or suppress the expression of cyclooxygenase-2 [24, 25], while glucosamine could oppose NFκB activation via O-GlcNAcylation of the anti-inflammatory protein A20 and decrease the production of inflammatory cytokines related to NF-kB activation [22, 26, 27]. In addition, glucosamine could mediate alternative macrophage activation in vivo and the production of leukotriene B(4) [28]. Previous evidence that androgens suppress leukotriene biosynthesis along with lower leukotriene production in males [25] suggested that anti-leukotriene therapy might be more relevant and effective for females, which was consistent with our findings that sex modified the inverse association between glucosamine use and gout. However, other mechanisms may be also involved and needed to be further examined.

Of note, diuretics are the well-known medications to be associated with gout, since diuretics could raise serum uric acid levels by increasing uric acid reabsorption and decreasing uric acid secretion in the kidneys. In our study, we observed a stronger inverse association of regular glucosamine use with risk of gout in participants with the usage of diuretics in females. It's biologically plausible considering that glucosamine could disrupt monosodium urate crystals-induced activation of the NLRP3 inflammasome by reducing mitochondrial reactive oxygen species generation inhibiting NLRP3 binding to PKR, NEK7 and ASC [1, 27, 29]. Taken together, glucosamine seems promising as a recommended protective agent for prevention of gout, especially for females taking diuretics.

The main advantages of this study include a large-scale population-based prospective study design with a long follow-up period, which shows the effectiveness of glucosamine supplementation in the real-life environment. However, several potential limitations should also be considered. First, it is difficult to distinguish the effects of a healthy lifestyle from habitual glucosamine use in this observational study. Although we carefully controlled for potential confounding factors and performed stratified analysis, the observed inverse associations might be driven by unmeasured or unknown health-related factors. Second, the lack of detailed information on the use of glucosamine supplementation, such as the dose, formulation (glucosamine sulfate, glucosamine hydrochloride, N-acetyl glucosamine), frequency and duration, precluded us from assessing the dose–response relationships of glucosamine supplementation and the appropriate duration of supplementation. Third, the participants were predominantly of European descent and healthier than the UK general population, which may limit generalizability of the findings to other populations. Owing to these limitations, further confirmation of the reported findings in future studies is necessary.

### Perspectives and significance

This large-scale prospective study found that glucosamine might be more relevant and effective for females and seems promising as a recommended protective agent for prevention of gout in female, but not in males, especially for those taking diuretics. Therefore, further clinical trials are needed to explore the effect of glucosamine supplementation on the occurrence of gout in females and encourage researchers to dissect the molecular mechanisms involved.

## Conclusions

In conclusion, this large-scale prospective study showed that regular glucosamine use is inversely associated with incident gout and the inverse association was modified by sex and diuretics use. These findings provide support that glucosamine may act as a potential supplementation for preventing gout in general population, and further clinical trials are needed to test this hypothesis.

## Supplementary Information

**Additional file 1: Figure S1.** Flow chart of study participants. **Figure S2.** Association of glucosamine use and the risk of gout stratified by potential risk factors in males. **Table S1.** Single nucleotide polymorphisms used to build the genetic risk score for gout.

## Data Availability

The UK Biobank data are available on application to the UK Biobank, and the analytic methods, and study materials that support the findings of this study will be available from the corresponding authors on request.
