# Supplementary material for: Associations of habitual glucosamine supplementation with incident gout: a large population based cohort study
Source: Biol Sex Differ. 2022 Sep 30;13:52. doi: 10.1186/s13293-022-00461-z (PMC9524004; doi:10.1186/s13293-022-00461-z)
Supplement: Supplementary file 1 — Additional file 1: Figure S1. Flow chart of study participants. Figure S2. Association of glucosamine use and the risk of gout stratified by potential risk factors in males. Table S1. Single nucleotide polymorphisms used to build the genetic risk score for gout. [file 13293_2022_461_MOESM1_ESM.docx]

**Additional Online Content**

**Figure S1. Flow chart of study participants.**

**Figure S2. Association of glucosamine use and the risk of gout stratified by potential risk factors in males.**

**Table S1. Single nucleotide Polymorphisms Used to Build the Genetic Risk Score for gout.**

502414 participants enrolled in the UK Biobank

495777 participants in the study

Excluded n=6637

Missing gout, n=863

Missing glucosamine supplement, n=5774

436594 participants finally included in this study

Excluded n=59183

Prevalent gout or using urate-lowering

medications, n=11057

Missing gout genetic risk score and

information of covariate, n=48126

**Figure S1. Flow chart of the participants in the current analysis**


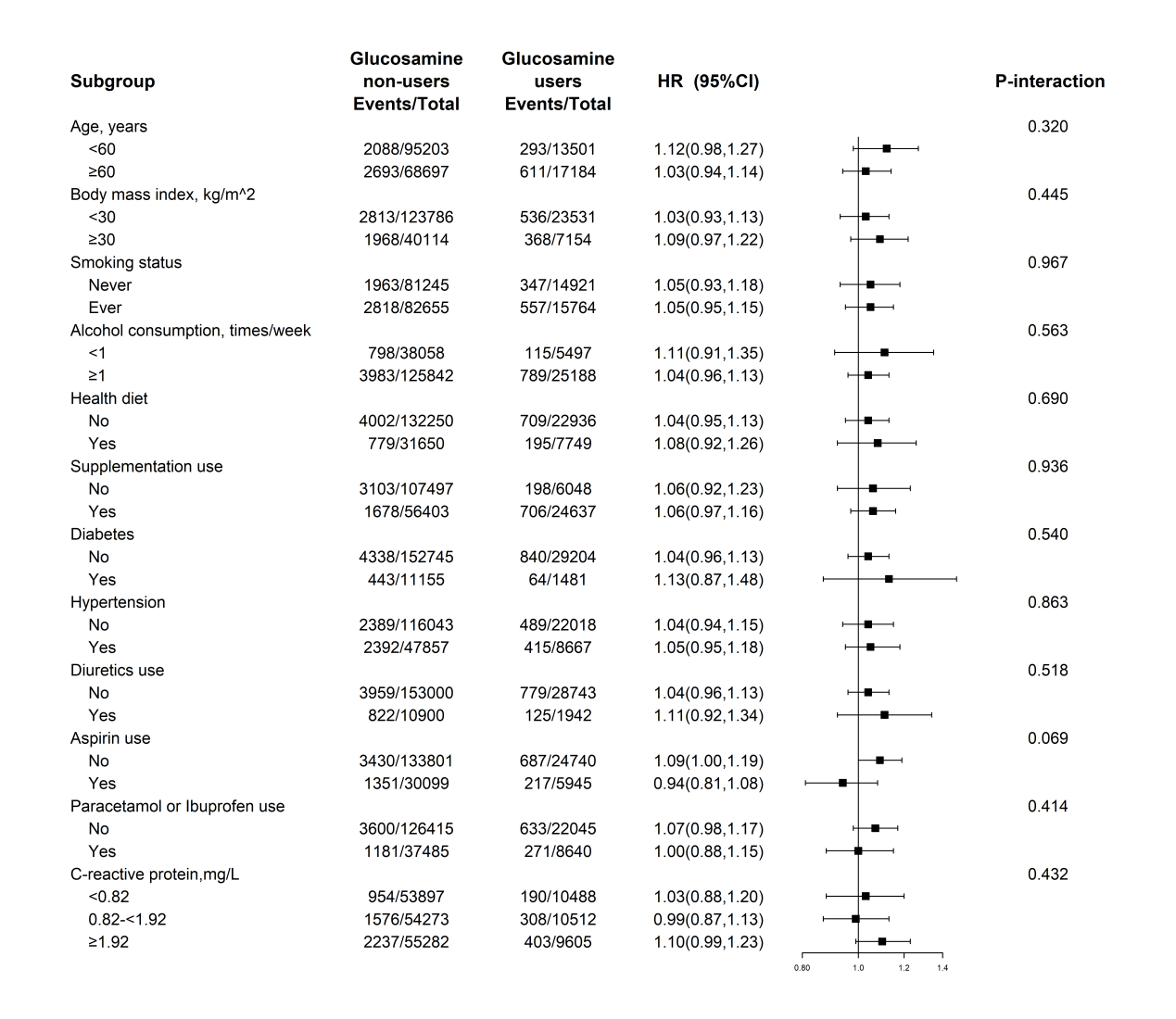


**Figure S2. Association of glucosamine use and the risk of gout stratified by potential risk factors in males. ***

* Results were adjusted for age, race, Townsend Deprivation Index, body mass index, smoking status, alcohol consumption, healthy diet score, vitamin or mineral supplementation, fish oil supplementation, comorbidities (hypertension, diabetes, high cholesterol, osteoarthritis, rheumatoid arthritis, and joint pain), drug use (cholesterol lowering medication, anti-hypertensive drug, insulin, aspirin, ibuprofen, paracetamol, and diuretics), estimated glomerular filtration rate and urate, if not already stratified.

**Table S1. Single nucleotide Polymorphisms Used to Build the Genetic Risk Score for gout.**

| **SNP** | **Chromosome** | **bp** | **Gene** | **A1** | **Beta** |
| --- | --- | --- | --- | --- | --- |
| rs1260326 | 2 | 27 730 940 | GCKR | T | 0.131 |
| rs2231142 | 4 | 89 052 323 | ABCG2 | T | 0.501 |
| rs13120400 | 4 | 89 033 527 | ABCG2 | C | -0.186 |
| rs7672194 | 4 | 89 126 647 | ABCG2 | T | 0.148 |
| rs4693211 | 4 | 89 249 061 | PPM1K- DT | C | 0.315 |
| rs28793136 | 4 | 89 216 768 | PPM2K- DT | C | 0.278 |
| rs1545207 | 4 | 89 239 492 | PPM3K- DT | A | 0.131 |
| rs16890979 | 4 | 9 922 167 | SLC2A9 | T | -0.261 |
| rs16891234 | 4 | 9 946 163 | SLC2A9 | C | 0.140 |
| rs1229984 | 4 | 100 239 319 | ADH1B | T | 0.399 |
| rs114791459 | 4 | 88 591 554 | LOC105377323 | A | 0.358 |
| rs114580333 | 4 | 88 790 118 | MEPE | A | 0.351 |
| rs2078267 | 11 | 64 334 114 | SLC22A11 | C | 0.140 |

SNP, Single nucleotide polymorphism.
